# Nutrition in Severe Dementia

**DOI:** 10.1155/2012/983056

**Published:** 2012-05-08

**Authors:** Glaucia Akiko Kamikado Pivi, Paulo Henrique Ferreira Bertolucci, Rodrigo Rizek Schultz

**Affiliations:** Behavior Neurology Section, Federal University of São Paulo (UNIFESP), 04025-000 São Paulo SP, Brazil

## Abstract

An increasing proportion of older adults with Alzheimer's disease or other dementias are now surviving to more advanced stages of the illness. Advanced dementia is associated with feeding problems, including difficulty in swallowing and respiratory diseases. Patients become incompetent to make decisions. As a result, complex situations may arise in which physicians and families decide whether artificial nutrition and hydration (ANH) is likely to be beneficial for the patient. The objective of this paper is to present methods for evaluating the nutritional status of patients with severe dementia as well as measures for the treatment of nutritional disorders, the use of vitamin and mineral supplementation, and indications for ANH and pharmacological therapy.

## 1. Introduction

Dementia is one of the most common and most significant health problems in the elderly and is one of the main causes of disability in older age [1] A growing proportion of older adults with Alzheimer disease (AD) or other dementias are now surviving to more advanced stages of the illness [2, 3].

Much clinical attention and research effort has been directed towards early diagnosis and mild stages of dementia, and prodromal stages, formerly classified as mild cognitive impairment (MCI). The later stages of the disease are as important as, if not more important than, the earlier stages because of the unique characteristics and events which occur affecting the lives of patients and their carers. Severe dementia (SD) is still relatively neglected and its prevalence is unclear, but it is estimated that one-third of dementia patients are in the severe stages [4–6].

There are several causes for concern in SD, when many impairments become more apparent: functional disabilities mean that carers have to take over more practical tasks, such as bathing, toileting, and feeding; there are special considerations with palliative care and ethical issues of terminal care along with physical problems [7–9].

SD, including AD, is associated with feeding problems, including difficulty in swallowing, leading to choking, chewing but failing to swallow, and active resistance to hand feeding. Because patients with dementia have limited language and communication abilities, it is difficult to identify the source of aversive behavior [9, 10]. Nevertheless eating and drinking may well be a sensory pleasure for people with SD. That potential source of pleasure should be maximized while at the same time ensuring optimum nutrition and hydration [11].

In addition, weight loss is a frequent complication of AD and occurs in patients at all stages, even in the early stages before diagnosis is possible. Malnutrition (namely, undernutrition) contributes to the alteration of general health status, to the frequency and gravity of complications, especially infections, and to a faster loss of independence. These states of malnutrition can be prevented or at least improved if an early intervention strategy is set up, but management must be rapid and appropriate. Weight loss is a phenomenon whose kinetics may vary; it can be a dramatic loss of several kilograms in a few months (severe weight loss) or a moderate but continuous loss as the disease progresses (progressive weight loss) [12, 13].

With progressive dementia, patients become incompetent to make decisions. As a result, complex situations may arise in which physicians and families decide whether artificial nutrition and hydration (ANH) is likely to be beneficial for the patient [14–16]. Many people with SD will ultimately experience dysphagia, which in turn is associated with aspiration pneumonia. Decisions about how to respond when someone develops swallowing problems are discussed elsewhere in this paper [17].

Artificial nutrition is the provision of liquid nutritional supplement by the enteral or parenteral route. The decision whether or not to provide artificial nutrition often evokes a powerful emotional response. Because surrogates and other loved ones agonize over the withholding and/or withdrawal of artificial nutrition, healthcare providers need to be ready to discuss the most current data regarding efficacy, complications, and the ethical and legal issues [18–20].

## 2. Definition of Severe Dementia

SD can be defined as the stage of the disease process of dementia in which cognitive deficits are of sufficient magnitude as to impair an otherwise healthy person's ability to independently perform the basic activities of daily life, such as dressing, bathing, and toileting. Definitions of SD vary from those which use a specific cutoff on a cognitive scale such as the Mini Mental State Examination. In accordance with the literature, we defined SD as follows [4]:

- score of less than 10 in the Mini Mental State Examination (MMSE) [21] or
- score of 3 or higher in the Clinical Dementia Rating (CDR) [22], or
- categories 6a to 7f in the Functional Assessment Staging Test (FAST) [23], or
- score of 6 or 7 on the Global Deterioration Scale (GDS) [24].

Some investigators state that the incorporation of these instruments into clinical trials has allowed the rate of worsening or improvement of patients to be quantified in advanced stages. Moreover, they have permitted better stratification of this long stage known as SD, which includes patients with very different cognitive, behavioral, and functional profiles. Thus, among patients classified as CDR 3, known as SD, there are individuals who still communicate verbally and walk without support, but also others who are confined to bed, unable to lift their heads, or already in the fetal position. The advantages of better classification of advanced dementia include the possibility of better studies designed to validate interventions in cognition, behavioral, and functional status, as well as better approaches in terminal dementia using palliative care [5, 25].

Although by definition the functional impairment in dementia must be related to the cognitive decline, there are a number of other factors such as parkinsonism and comorbid disorders that by themselves contribute significantly to the functional disability [26].

So, unlike the dying trajectory in more-acute illnesses, patients with dementia have a long period of severe functional and cognitive impairment before death [27].

## 3. Nutritional Evaluation of Patients with Advanced Dementia

Cachexia and weight loss are common signs among AD patients. These findings have been studied to see if there is any correlation between organic deficiency caused by low-energy intake and the acute state of hypercatabolism these patients present. Undernourishment has been suggested to be a factor in the etiology of dementia and other psychiatric, and cognitive disorders, although nothing has been proven in this respect [28, 29].

Weight loss in AD may be correlated to the presence of higher rates of infection, the burning of energy due to repetitive movements and cognitive deficit that compromises the patient's independence [30, 31].

This process increases the risk of infections, skin ulcers and stimulates loss of body temperature, which consequently compromises the quality of life of patients with AD [32].

There is a theory behind the weight loss seen in AD patients that is based on the morphology and that has to do with the brain lesions caused by the disease, and a significant association has been found between low body weight and atrophy of the mesial temporal cortex in the region of the central nervous system responsible for eating behavior [33].

Guérin et al. [12] observed two forms of weight loss, one slow and progressive, the other rapid and severe. A severe weight loss was related to the seriousness of the disease, the higher number of hospital admissions, clinical events, and institutionalization, which led the researchers to conclude that these patients might benefit from nutritional interventions.

Munõz et al. [34] found that there is impairment of the energy and muscle reserves at all stages of the disease, but that there is a greater impairment of the Body Mass Index (BMI), and brachial fatty and lean mass, in the advanced stage.

Given these findings, evaluation of their nutritional status is crucially important for these patients. The result of the nutritional evaluation will guide the management to be followed in each case.

The nutritional evaluation of these patients may be carried out using a number of traditional methods based on objective evaluations such as anthropometry, the evaluation of clinical signs that are indicative of malnutrition, evaluation of food intake, the subjective Mini Nutritional Assessment (MNA), and by NSI-Nutrition Screening Initiative [35].

The MNA was developed in order to assess the risk of malnutrition in the elderly and identify those who could benefit from early intervention. This evaluation is made of 18 items including: anthropometry, nutritional evaluation, global clinical evaluation and self-perception of health, and may be used both in screening and to evaluate the nutritional status; any trained health professional can apply it [36].

The NSI is a self-applied questionnaire made up of 10 questions, created in order to assess primary health care; however, its use is not widely recommended since it has shown limited power of prediction for mortality in the elderly [37].

 Most authors, despite these methods, consider the use of anthropometrical measurements to be the gold standard for nutritional evaluation, along with outpatient tests for total lymphocytes, albumin, blood cholesterol, hemoglobin, and transferrin [38].

A nutritional evaluation study made in a long-stay institution with dementia syndrome, depression, and cardiovascular diseases, using anthropometric data gathering, found that 36.6% of these patients were malnourished, demonstrating the importance of early nutritional intervention [39].

Spaccavento et al. [13], using MNA to investigate the role of the nutritional status, correlating it to cognitive, functional, and neuropsychiatric deficits in AD patients, found that patients at risk of malnutrition showed greater impairment, both in simple instrumental daily life activities (DLA and IDLA) and a greater likelihood of presenting hallucinations, apathy, aberrant and nocturnal motor behavior on the sub-scales of the Neuropsychiatric Index (NPI).

Weight loss has a negative impact on the prognosis of AD since the more severe the malnutrition is the faster will be the clinical progression leading to the death of patients [40].

## 4. Nutritional Management of Patients with Advanced Dementia

### 4.1. Does Oral Nutritional Therapy (ONT) in Patients with Advanced Dementia Bring Benefit for the Nutritional Status?

In patients with advanced dementia there is a reduced capacity for communication, loss of pleasure in eating, changes in mastication leading to difficulty in swallowing certain consistencies of food, and culminating in dysphagia [41].

These patients tend to present reduced mobility leading to loss of muscle mass even when not malnourished. In these cases nutritional therapy plays an essential role since it aims to provide comfort in the eating process and assure the patient's dignity [31].

As AD advances, the difficulty of keeping these patients' weight up through conventional feeding increases, and in these cases it is recommended to use high-calorie concentrates [42].

ESPEN, the European Society for Clinical Nutrition and Metabolism [43], classifies as evidence level C the use of dietary supplements to maintain the nutritional status of patients with dementia.

Pivi et al. [44], in their study of oral nutritional therapy with patients at different stages of AD, found that an additional 690 kcal/day in these patients' diets led to an improvement of the nutritional indices BMI, BC (brachial circumference), BMC (brachial muscle circumference), and TLC (total lymphocyte count), demonstrating that nutritional supplementation is in fact effective at any stage of the disease.

Carver and Dobson [45] likewise found that dietary supplementation significantly increases body weight, tricipital skin fold, and brachial muscle circumference in hospitalized elderly dementia patients.

Although dietary supplementation shows benefits for nutritional status, only 11% of outpatients use them, as a study by Trelis and López [46] showed. This study highlighted the importance of the entire team involved in the treatment of dementia patients being able to perceive the onset of nutritional deficit and so refer the patient to the nutritionist to indicate the best type of supplement for each case.

Other studies with nondemented elderly patients with other clinical needs also showed the effectiveness of using dietary supplements in addition to the habitual diet. Volkert et al. [47] showed that elderly patients over 75 who were given calorific supplementation of 500 kcal/day improved their convalescence and recovery from deficiency states.

It is important to mention that there are lines of research that recommend the prescription of dietary supplements only for patients with low BMIs, since they may abandon their habitual diets in favor of dietary supplements [48].

Young et al. [49] also demonstrated this concern in a study with use of dietary supplements for 21 days in addition to their habitual diets. Those patients who received nutritional supplements were observed to have less likelihood of increasing energy intake in their habitual diets. The researchers stressed out that patients with low body weight have a greater chance of benefitting from the use of nutritional supplements.

This whole discussion leads us to observe that a natural diet ingested by mouth promotes patient comfort and dignity and may not shorten survival, since there are reports of patients who survived two years exclusively feeding by mouth [31]. There is evidence that dietary supplementation may enhance the nutritional status of dementia patients, regardless of the phase at which they are. The significance of this nutritional improvement has yet to be determined for the rate of cognitive and functional decline.

## 5. Use of Vitamins and Minerals in Alzheimer's Patients

No specific or definitive environmental risk factor has been identified as the likely cause of AD, but there are research lines that correlate diet as a preventive and improving factor for the dementia states.

There is evidence of worsening of the cognitive condition linked to the increase of homocysteine, whereas the consumption of fats and red grapes seems to have a protective effect for AD patients [50].

De Jong et al. [51] studied patients who followed dietary instructions and consumed foods supplemented daily with 0.25 mg of folic acid and 2.5 mg B6 vitamin; they found that these participants showed a reduction in the serum concentration of homocysteine of around 25%. This result suggests that a low concentration of homocysteine may inhibit the development of dementia.

Morris et al. [52] studied nurses who consumed polyunsaturated fatty acids at least once a week; although results were not conclusive, they suggested that this cohort had a lower risk of developing AD.

Polyphenols, in particular resveratrol, found in great quantities in red grapes, are believed to reduce the incidence of AD owing to their antioxidant properties, although this has not yet been proved [53, 54].

A study of supplementation with low concentration of folic acid and B vitamin complex were made to investigate the association of these substances with enhanced cognitive function. It raised the prospect of reduction of dementia risk, but researchers emphasized that more studies are needed to prove this [55].

Study with AD patients in the mild phase who received micronutrient supplementation for six months found that, when compared to the control group, there was no benefit in the supplementation for the evolution of the disease, the nutritional status, the biochemical parameters, the cognitive function, and the behavior and eating disorders that these patients commonly present [56]. 

Despite the evidences, caution must be taken in adopting such approaches, since more studies on micronutrient supplementation need to be performed in order to observe real benefits for dementia.

## 6. Indication of Artificial Nutrition and Hydration in Advanced Dementia Patients

The use of Enteral Nutrition Therapy (ENT) or Artificial Nutrition and Hydration (ANH) is only indicated when there is a risk of malnutrition and severe impairment of the swallowing process, with the possible consequence of aspiration pneumonia [57].

According to ASPEN—American Society for Parenteral and Enteral Nutrition [58], an ENT in patients with advanced dementia is classified as category E, not obligatory in those cases. The decision for ENT must be based on effective communication with the caregivers to decide whether it should be used or not.

The use of nasogastric or nasoenteric tubes in patients with dementia is not recommended as they may pull out the tube, leading to discomfort for patient and family both, since the patient will have to be reintubated [59]. Gastrostomy is normally indicated as the route for alternative feeding, since it is already commonplace and laid down in rules when the duration of ENT is over six weeks [60].

ANH using feeding tubes or gastrostomy has been routinely indicated for patients in advanced stage of dementia who present serious problems swallowing, in order to prevent malnutrition, hydrate the patient properly, and provide comfort. Some studies, however, have put forward issues for the nonuse of ANH in these patients [59].

See Table 1 setting out the major studies in this field and their recommendations regarding ANH in advanced dementia patients:

The use of ANH in patients with dementia is very controversial being necessary identification of the goals for an intelligent and rational judgment [65].

Deciding whether to use ANH in dementia patients is a challenge and many caregivers may take their decisions without adequate information and based on an over-optimistic view of the future clinical course of this patient. Health professionals, relatives, and patients should be aware of the realistic expectations of tube feeding and of its risks and benefits before taking such a decision [18].

Ethical dilemmas are related to ANH and it is useful in cases of severe dementia, being necessary to make valid clinical judgments and to guide patients and their families to exchange options related to initiating, withholding, or withdrawing ANH. All this process should be comprehensive and understood from theory to practice. The use of informed consent for competent caregivers is important in those cases [66].

## 7. Pharmacologic Therapies

Several randomized controlled trials have been published on atypical antipsychotic therapy for behavioral and psychological symptoms in patients with SD.

Some results suggested no significant difference between any of the treatments compared with placebo. They also support the recommendation for the use of atypical antipsychotics only in the presence of severe agitation, aggression, or psychosis that places the patient or those in their environment at risk. Other psychotropic drugs include the antidepressants and anticonvulsants. On balance, the efficacy of the atypical antipsychotics appears to be superior to that of other classes of drugs despite the increased risks. However, there are few, if any, head-to-head comparisons to truly characterize all risks and benefits [7]. In a meta-analysis adverse events of atypical antipsychotics were mainly somnolence and urinary tract infection or incontinence across drugs, and extrapyramidal symptoms or abnormal gait were observed with risperidone or olanzapine. There was no evidence for increased injury, falls, or syncope. But there was a significant risk for cerebrovascular events, especially with risperidone [67]. With all these studies it is possible to imagine that management of behavioral disorders could improve nutritional status. We also know that some antipsychotic drugs could improve appetite, and we could use these drugs to improve food intake. But more studies are needed to better assess clinical significance and effectiveness of these hypotheses.

Pharmacological therapies for the improvement of cognition include cholinesterase inhibitors and memantine, suggesting that this class of medication improves cognition, function, behavior, and global measures. There is a consensus to recommend their use in patients with severe Alzheimer's disease.

The most common side effects are gastrointestinal and include anorexia, nausea, vomiting, and diarrhea [7]. It is therefore very important to pay attention to these problems so as not to impair food intake. There are no studies in the literature to demonstrate whether cholinesterase inhibitors or memantine could improve the nutritional status.

It is also important to register that there are no well-designed randomized studies to demonstrate benefits of appetite stimulant drugs. We know that it is common medical practice, but evidence of the mechanisms, safety, and efficacy is lacking.

## 8. Final Remarks

There have been studies to develop drugs and treatments to understand nonpharmacological measures, to find epidemiological factors that affect the natural history of this disease, but they are not conclusive to define ways to approach the end of life or to look into issues such as suitable nutrition for these patients. The long drawn-out course and uncertain time frame of the prognosis raise considerable doubts as to decisions to be taken by the team of carers and by the relatives.

This paper demonstrates the palliative care that should be given in order to improve the patient's quality of life. All aspects of the treatment of these patients should therefore be part of the responsibilities of the multiprofessional team. It should act cohesively to help relatives take the best decisions in choosing treatment for their loved ones.

This view of comfort and of the right proportions between actions taken and the principle of nonharm may serve as a guide in decision making, especially as regards nutrition issues [8].

## Figures and Tables

**Table 1:** Results of the main studies in artificial nutrition and hydration (ANH) in patients with severe dementia.

Study	Objective and methodology	Results	Conclusion
Ciocon et al. [61]	Evaluate the indications, benefits, and complications of ANH in patients using enteral tube, with serious swallowing disorders and at risk of aspiration	67% of patients were agitated when the tube was removed and 43% had aspiration pneumonia during the study	Researchers concluded that the use of ANH may lead to a high frequency of complications

Finucane et al. [10]	Review of the literature (1966 to 1999) to evaluate the use of ANH in advanced dementia	No data to suggest that the use of ANH may prevent aspiration pneumonia, reduce the risk of infections or prolong life	Most studies show that there are substantial risks in using ANH in these patients, and thus its use is discouraged

Li [62]	Review the use of ANH in patients with advanced dementia	ANH does not prevent malnutrition, reduce the occurrence of bed sores, or prevent aspiration pneumonia, nor does it prolong life	Although it may not be effective in preventing malnutrition and dehydration, this type of feeding allows a degree of maintenance and patient comfort

Pasman et al. [19]	Evaluate the discomfort of patients with serious dementia of a Dutch nursing home	The highest rates of discomfort observed in these patients were in dyspnea, acute pain, and agitation	Not choosing ANH did not correlate with the level of discomfort of the patients. ANH was identified as an individual decision

Clarfield et al. [63]	Evaluate the use of ANH in patients with advanced dementia admitted to Israeli and Canadian hospitals in order to find the main differences between ethnicities and ethical issues	24,5% patients were in if fed by ANH	These results may be explained by a combination of administrative or financial incentives and by religion and culture

Volkert et al. [43]	ESPEN Guideline developed to recommend use of ONT or ANH in elderly patients based on scientific evidence	No increase in survival associated with this use, leading the researchers to consider ANH only according to patient's or family's wishes	ANH not recommended in patients with advanced dementia

Buiting et al. [64]	To investigate the opinion of Dutch and Australian physicians on use of ANH in patients with advanced dementia	Dutch physicians based their decision on a wide-ranging evaluation, while Australian physicians tended to use scientific evidence	All are reluctant at beginning of ANH but Dutch physicians tend to take primary responsibility for the decision, while Australian physicians prefer to leave the decision to the family
